# Diet shapes the gut microbiome of pigs during nursing and weaning

**DOI:** 10.1186/s40168-015-0091-8

**Published:** 2015-07-01

**Authors:** Steven A. Frese, Kent Parker, C. Chris Calvert, David A. Mills

**Affiliations:** Department of Food Science and Technology, University of California Davis, Davis, CA 95616 USA; Foods for Health Institute, University of California Davis, Davis, CA 95616 USA; Department of Animal Science, University of California Davis, Davis, CA 95616 USA

## Abstract

**Background:**

The newborn mammal is rapidly colonized by a complex microbial community, whose importance for host health is becoming increasingly clear. Understanding the forces that shape the early community, especially during the nursing period, is critical to gain insight into how this consortium of microbes is assembled. Pigs present an attractive model for nursing humans, given physiological and compositional similarity of pig and human milk and the utility of pigs in experimental studies. However, there is a paucity of data examining the gut microbiome in nursing pigs from birth through weaning using modern molecular methods and fewer experimental studies that examine the impact of diet on these microbial communities.

**Results:**

We characterized the fecal microbiome of pigs from birth through 7 weeks of age, during which the animals were transitioned from an exclusive diet of sow milk to a starter diet composed of plant and animal-based components. Microbial communities were clearly distinguishable based on diet, being relatively stable absent dietary changes. Metagenomic sequencing was used to characterize a subset of animals before and after weaning, which identified glycan degradation pathways differing significantly between diets. Predicted enzymes active on milk-derived glycans that are otherwise indigestible to the host animal were enriched in the microbial metagenome of milk-fed animals. In contrast, the bacterial metagenome of weaned animals was enriched in functional pathways involved in plant glycan deconstruction and consumption.

**Conclusions:**

The gut microbiome in young pigs is dramatically shaped by the composition of dietary glycans, reflected by the different functional capacities of the microbiome before and after weaning.

**Electronic supplementary material:**

The online version of this article (doi:10.1186/s40168-015-0091-8) contains supplementary material, which is available to authorized users.

## Background

The mammalian gastrointestinal tract is a home to a complex and diverse microbial community that profoundly influences health and disease. The communities that assemble in this ecosystem, beginning shortly after birth, have been shown to be remarkably host-specific and broadly stable over time [1, 2]. Studies of the gut microbial community illustrate how populations of constituents are shaped by environmental exposure to microbes, diet, immunological pressures, host genetics, and ecological forces within the ecosystem itself [3–5]. The dramatic importance of this community for host health and resistance to disease has led to the evolution of elaborate mechanisms to facilitate the acquisition of this community across generations.

In shaping these populations, milk has been shown to provide an important selective advantage to some microbes, allowing them to dominate an ecosystem [6–8]. This has been best described for humans, but the secreted glycans found in human milk (human milk oligosaccharides (HMOs)) are structurally similar to those found in bovine and porcine milks [9, 10]. Understanding which microbial populations are influenced by milk and their fate at weaning provides insight into how unique, and powerful, dietary changes in the lives of mammals shape the gut microbiome. It has been hypothesized that milk, especially milk glycans, have a controlling effect on the microbiome of nursing mammals, resulting in a “milk-oriented microbiome” (MOM) [11]. However, it is poorly understood how exposure and diet interact to shape the microbiome, that is, whether stochastic exposure or the diet, in this case milk, drives the major populations and changes observed in the microbiome early in life.

Few studies examine the microbiome of nursing pigs, and no current studies follow animals from birth past weaning longitudinally. In this study, we followed the microbial communities in nursing pigs from birth through weaning, up to 7 weeks of age. Using 16S rRNA marker gene sequencing, we found that diet, rather than litter effects or age, best described the differences between samples. Metagenomic sequencing of microbial communities confirmed this distinction, discriminating samples starkly by functional capacities related to dietary glycans, found in the animal’s respective diets. By the end of our study, the fecal microbiome of animals studied here closely resembled that of age-matched animals in other studies, suggesting that early stochasticity is replaced by later convergence and shaped by diet [12–15].

## Results

### Community diversity increases over time but populations shift with diet

**Fig. 1:**
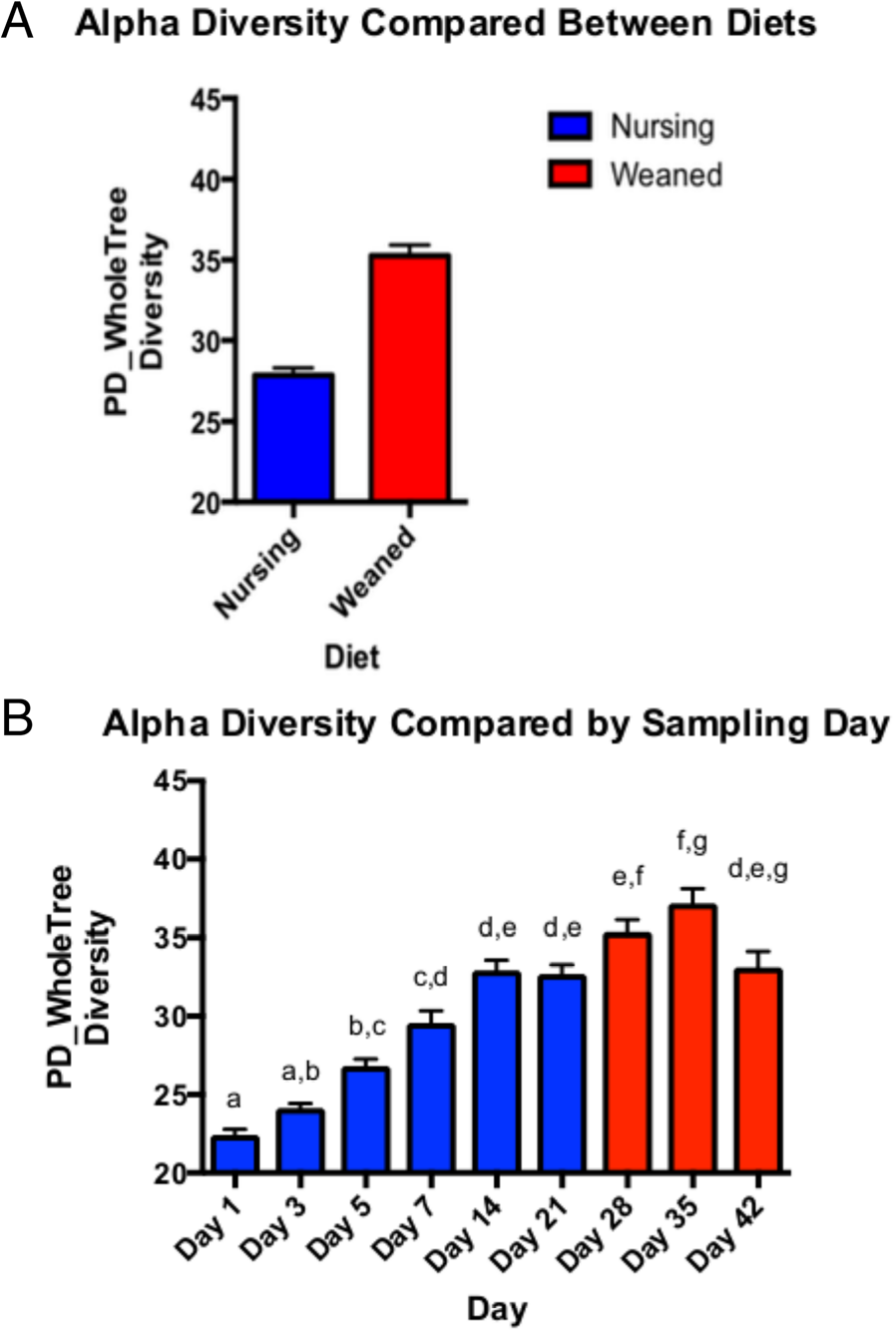
Pig fecal alpha diversity (phylogenetic distance, PD), measured in rarified samples, compared between diets (**a**) and over time (**b**). *Bars* are colored by diet, nursing (*blue*) or weaned (*red*). *Bars* are shown ± SEM

16S rRNA sequencing produced 2,966,033 reads after quality-filtering, giving a mean sample depth of 13,067 reads with a standard deviation of 7,159 reads. Alpha diversity analysis showed a stark contrast between nursing and weaning animals (Fig. 1a, *p* < 0.001), but when examined over time, a gradual increase in alpha diversity (phylogenetic distance) from birth through 21 days is observed. This trend plateaued after day 21 through the rest of the time points studied (Fig. 1b), but differences between litters were not significant (*p* > 0.05).

**Fig. 2:**
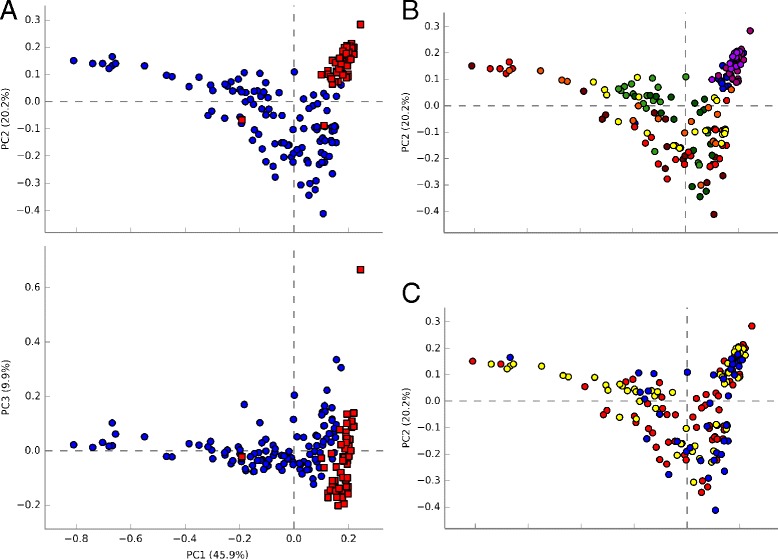
Principal component analysis plots of pig fecal microbiota colored by diet (**a** nursing, *blue*; weaned, *red*), day (**b** day 1, *dark red*; day 3, *red*; day 5, *orange*; day 7, *yellow*; day 14, *green*; day 21, *dark green*; day 28, *blue*; day 35, *dark blue*; day 42, *purple*), or litter (**c** litter 1, *red*; litter 2, *yellow*; litter 3, *blue*)

Similarly, bacterial community composition was not significantly different between animals when measured by ANOSIM distances (*p* > 0.05, *R* = 0.0182), which compares community composition, wherein identical communities are given an *R* statistic near 0, and completely distinct communities are given a value of +1. While very small, but significant, litter effects were observed (*p* = 0.032, *R* = 0.0311); time described differences between samples more robustly (*p* < 0.001, *R* = 0.4786). However, it was diet (nursing compared to weaned, *R* = 0.6899, *p* < 0.001) that best explained sample distances. These differences were recapitulated in PCA plots of the data which explained 76 % of total variation in three primary principal axes and show clear grouping based on diet, more so than when visualized by sample day (Fig. 2a–c).

**Fig. 3:**
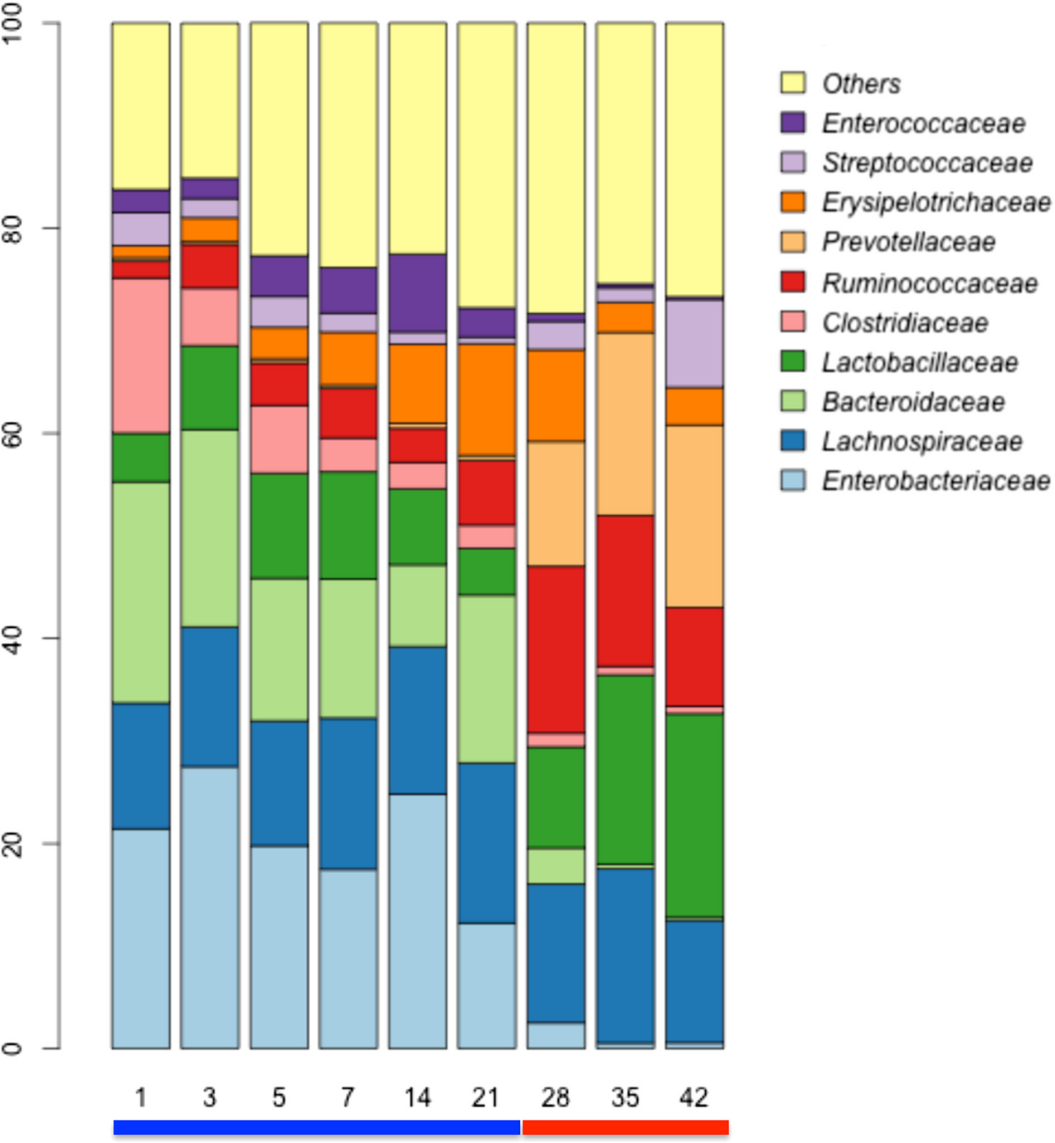
Stacked bar plots showing average percentage of bacterial populations in pig feces over time, from left to right, at day 1, 3, 5, 7, 14, 21, 28, 35, and 42. Colored bars below plot indicate diet (*blue*, nursing; *red*, weaned)

**Fig. 4:**
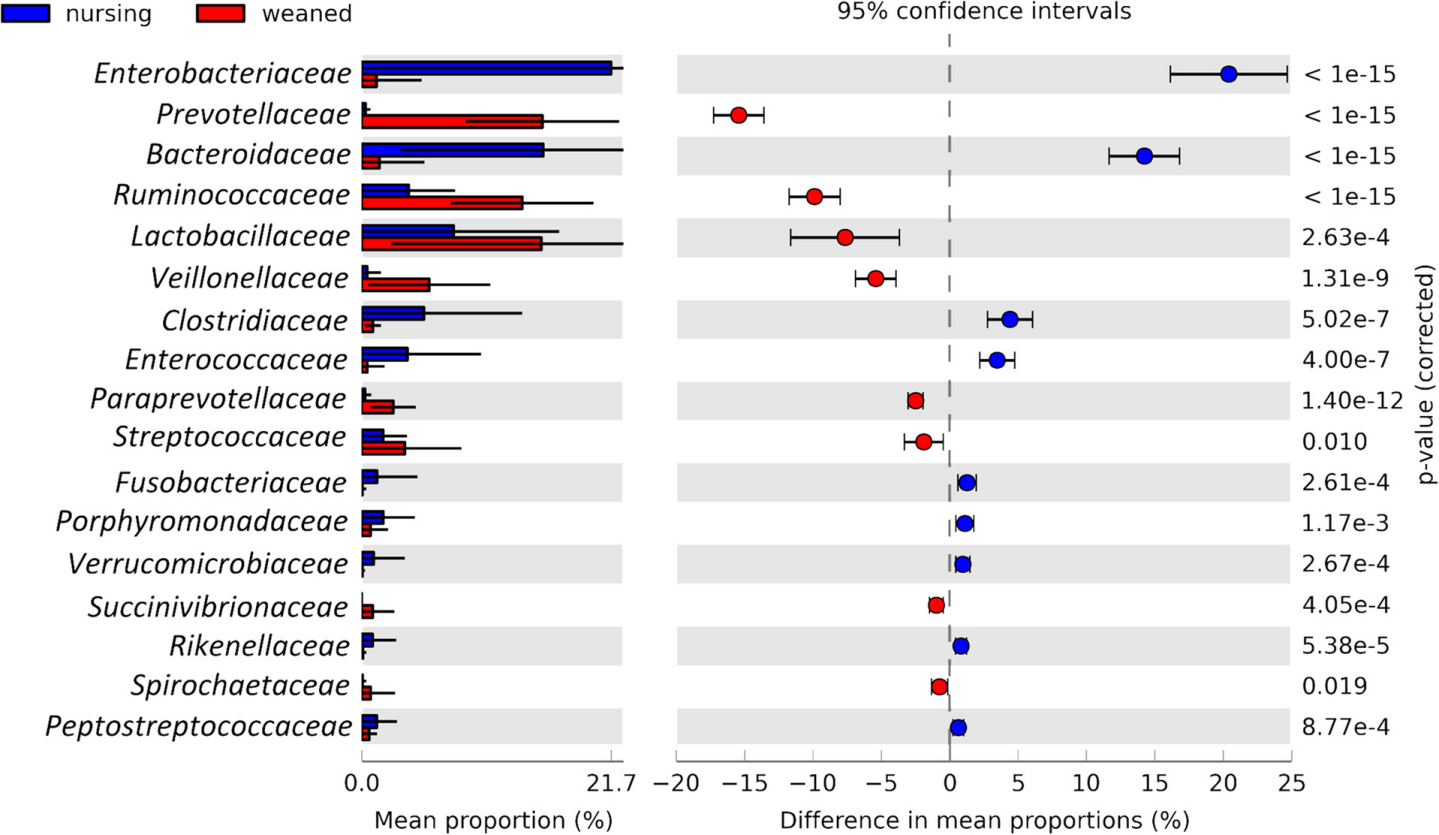
Extended error bar plot identifying significant differences between mean proportions of bacterial taxa in nursing (*blue*) and weaned (*red*) samples. Corrected *p* values are shown at right

Surprisingly, the microbial communities in nursing pigs were relatively stable at the family level from at least day 1–21, with the more abundant populations of *Bacteroidaceae*, *Clostridiaceae*, *Lachnospiraceae*, *Lactobacillaceae*, and *Enterobacteriaceae* composing a majority of the community throughout this time (Fig. 3). After weaning (day 28 and beyond), populations of *Bacteroides* and *Enterobacteriaceae* declined and populations of *Lactobacillaceae*, *Ruminococcaceae*, *Veillonellaceae*, and *Prevotellaceae* increased. When comparing the nursing to weaned microbiota, significant differences were apparent (Fig. 3). First, *Prevotellaceae* increased nearly 50-fold from an average 0.3 % in nursing animals to 14.8 % in weaned animals (Fig. 3, Additional file 1: Figure S1). This coincided with a decrease in the population of *Bacteroidaceae* from 15.4 % in nursing animals to 1.4 % in weaned pigs (on average). There is also an increase in *Ruminococcaceae* from 1.7 to 9.6 % after weaning. *Lactobacillaceae* increased over time from 5.5 to 19.1 % after weaning. In contrast, *Lachnospiraceae* did not change dramatically throughout the study, ranging from 4.6 to 9.1 % of total abundance. Taxonomic changes that differed significantly between each diet are shown in Fig. 4.

### Functional composition of the metagenome reshaped by diet

**Fig. 5:**
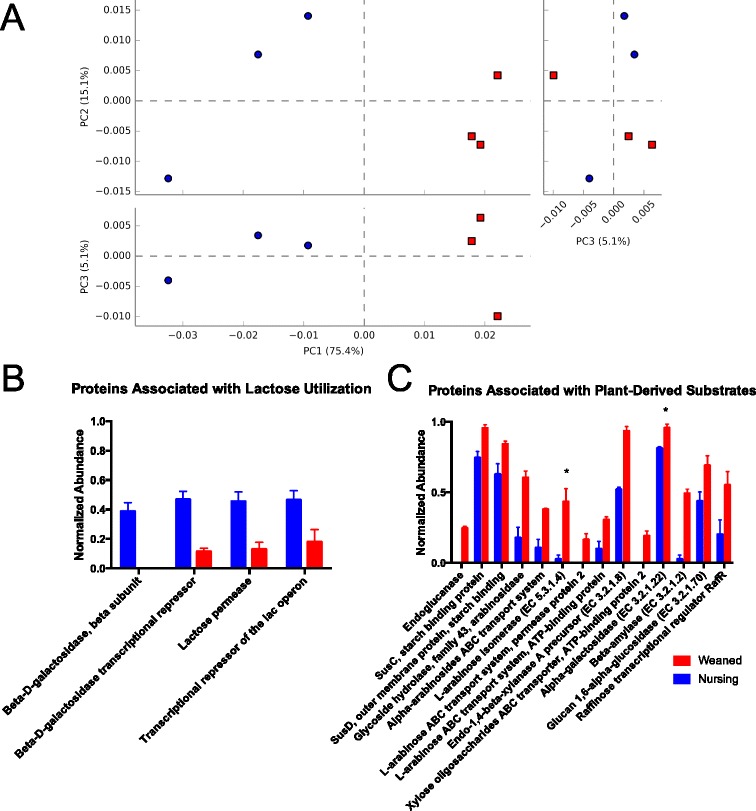
Metagenomic sequencing of pig fecal samples analyzed using PCA (**a**), and bar charts comparing normalized abundances of individual predicted genes involved in lactose utilization (**b**) and plant-derived glycans (**c**). All differences between diets shown (*blue*, nursing; *red*, weaned) were significant (*p* < 0.01), unless indicated with an *asterisk*, where *p* < 0.05

MGRAST classification of shotgun metagenome sequencing reads resulted in an average of 4.97 million reads per sample, after quality control and filtering of host DNA reads, for reads that encoded predicted proteins with known functions. Samples were compared by PCA, based on normalized read abundances, using reads that could be mapped to a functional annotation. PCA clustered the two diets (nursing vs. weaned) distinctly, with three primary PC axes describing 95.6 % of total variation, of which 75.4 % of variation was in the first principal axis (Fig. 5a). Of note, chief differences between samples were reads that were functionally annotated as being involved in carbohydrate metabolism (Fig. 5b, c). Of particular interest, samples from nursing animals were significantly enriched for genes mapping to lactose catabolism, *N*-acetylglucosamine catabolism, galactose/galactonate catabolism, and sialic acid catabolism (Fig. 5b, Additional file 2: Figure S2A). Reads mapping to starch, β-glucan, xylose, and arabinose-degrading enzymes were significantly enriched among samples from weaned animals (Fig. 5c, Additional file 2: Figure S2B). In fact, whole catabolic pathways for dietary glycans (hydrolases, transporters, catabolic pathways, regulators) were enriched relative to their abundance in each of the diets (Fig. 5, Additional file 2: Figure S2).

**Fig. 6:**
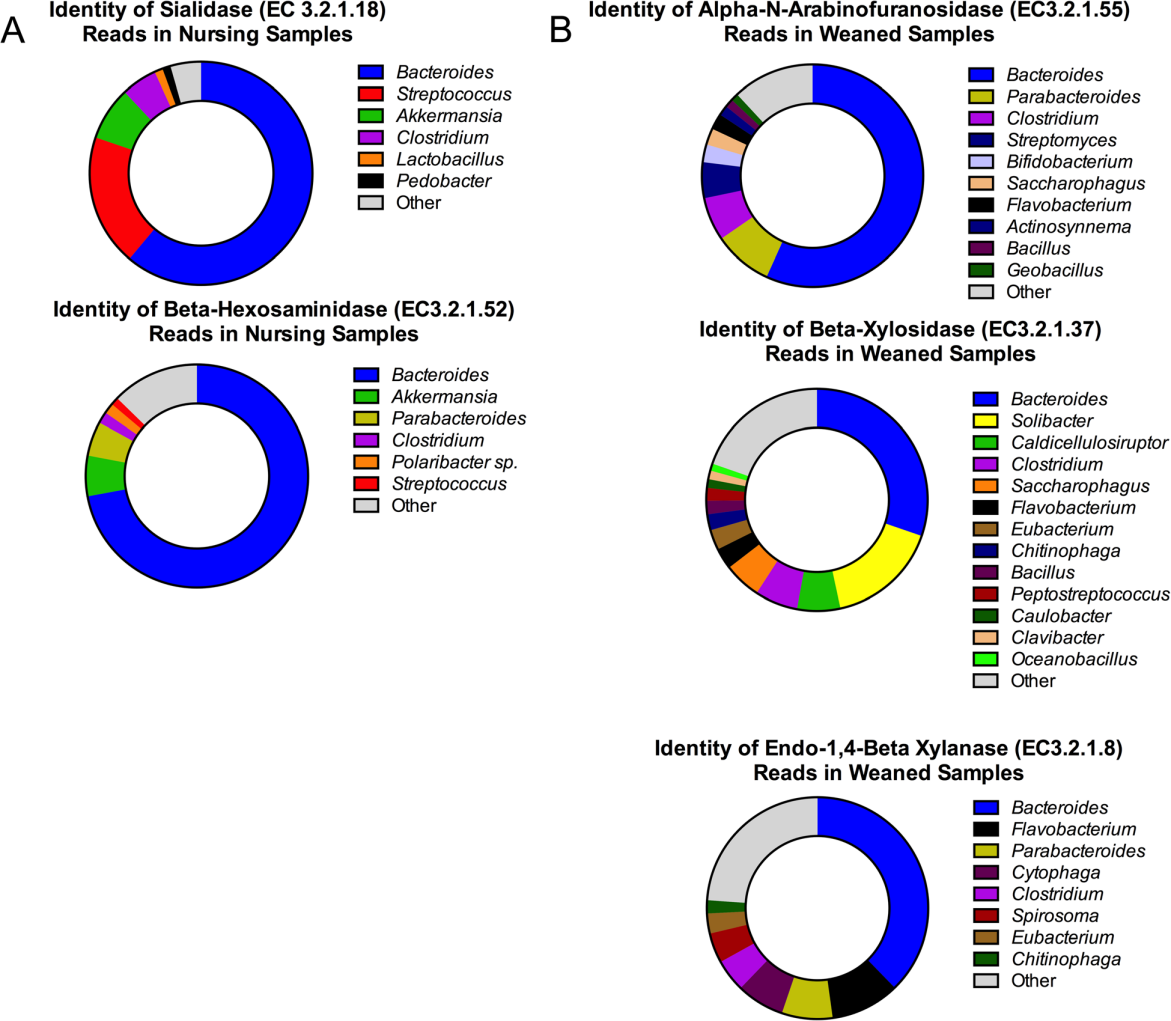
Distribution of metagenomic reads from pig fecal samples annotated as key catabolic enzymes in (**a**) nursing or (**b**) weaned animals

Changes in key catabolic steps, notably the hydrolysis of dietary glycans, were found to be the result in changes in taxa significantly associated with each diet. Key steps in the breakdown of complex milk glycans, by the action of sialidases (EC3.2.1.18), β-hexosaminidases (EC 3.2.1.52) were contributed primarily by *Bacteroides* (Fig. 6a). In weaned animals, β-xylosidases (EC 3.2.1.37), endo-1,4-β-xylanases (EC 3.2.1.8), and α-*N*-arabinofuranosidases (EC 3.2.1.55) were more evenly distributed among a broader diversity of taxa (Fig. 6b).

## Discussion

### The microbiome is rapidly assembled in pigs

Through exposure after parturition, the gut microbiome develops rapidly in a mammal’s early life. Large taxonomic shifts have been observed in humans through the first years of life [16–19], and here we report that microbial turnover in pigs early in life is also dramatic as the animals shift to an adult-like diet. Unlike humans, the weaning process for pigs is an abrupt dietary shift, from solely sow milk to a complete feed based diet, that produces similarly abrupt taxonomic and functional shifts in the gut microbiome (Fig. 3). While there is wide variation, human infants are generally introduced to novel foods gradually, the result of which is rapidly reflected in the gut microbiome [18]. Interestingly, early colonization of these animals did not include a “succession” of taxa, as has been reported for early human colonizers, where *Lactobacillus* or *Proteobacteria* are early, transient colonizers and are supplanted by more stable populations of *Bifidobacterium* or *Bacteroides* [20–22]. Instead, in pigs, we observe dominant, stable populations from the first day after birth whose principle composition remain for the first 3 weeks of life, while minor populations begin to colonize, increasing overall diversity over time (Figs. 1 and 3). While in humans, much attention has focused on western populations and how delivery mode shapes the infant gut microbiome, some human populations show a much less dramatic effect of delivery mode [23]. Our results in pigs suggest that exposure to these microbes may seed these early colonizers, who are present in low abundances but unable to consume available substrates, rather than a coordinated ecological succession during nursing.

In these animals, the nursing period is clearly dominated by a milk-oriented microbiome; a community whose functional genetic content reflects its focus on the consumption of milk (Fig. 4b, Additional file 2: Figure S2A). Other recent studies on pigs have found comparable trends, composed of the same taxa, suggesting the broad applicability of these results among animals in the US, South Korea, and France [13, 15]. For example, one of the most dramatic changes observed was in a small population of *Prevotella* that is present in nursing animals from birth. This population is very low in relative abundance and increases rapidly and dramatically when a more favorable diet is introduced at weaning (Fig. 3, Additional file 1: Figure S1). This genus is widely associated with plant polysaccharide consumption and is enriched by plant polysaccharides [24], which are found in the diet after weaning but not in sow milk and typifies the dramatic taxonomic shifts associated with weaning. Indeed, these *Prevotellaceae* appear to supplant *Bacteroides* populations in nursing pigs, whose ability to harvest milk glycans is well characterized [25, 26] and has been hypothesized to be linked to the dietary dichotomy we describe here [13].

### The milk-oriented microbiome harvests dietary milk glycans

Milk glycans are a structurally and compositionally diverse and abundant carbon source in milk, best described for humans [27], but present in the milk of many different mammals [10]. It has been suggested that these glycans have a profound impact on the gut microbiome, shaping a composition described as the “milk-oriented microbiome” or “MOM” which contributes to neonatal health [11]. Porcine milk is composed primarily of *N*-acetylglucosamine, sialic acids (*N*-acetylneuraminic or *N*-glycolneuraminic acid), galactose and glucose monomers, and less abundantly, fucose. These monomers are linked together to form trimers, tetramers, and more complex bi-antennary structures [9]. While structurally less diverse than human milk oligosaccharides, these compounds are compositionally similar to both human and bovine milk oligosaccharides and require a similar array of enzymatic capacities to deconstruct these structures. As the host lacks the metabolic capacity to consume these highly sialylated complex carbohydrates, they pass undigested to the distal GIT, where they shape the composition of the gut microbiome. As the host digests and absorbs simple sugars proximally to the distal gut, only glycans resistant to digestion pass through to influence the distal gut microbiota. In this study, we found that the enzymatic capacities necessary to remove and deconstruct milk sugar components were enriched, relative to the community found in weaned animals, where enzymes predicted to encode plant glycan hydrolases were increased (Fig. 4c, Additional file 2: Figure S2B). This implies also that mucus glycans, which are structurally similar to milk glycans, play a very minor role, if any, in shaping the gut microbiome of weaned pigs. Further, metabolic pathways for the catabolism of galactose, lactose, *N*-acetylglucosamine, and sialic acid were also enriched (Additional file 2: Figure S2A), suggesting that the gut microbiome of nursing pigs is both enzymatically and metabolically oriented to the consumption of milk oligosaccharides, as is the case for infant-associated microbes in humans [8].

### The weaned microbiota harvest plant glycans

The abrupt weaning methods used in pig rearing allow for an excellent experimental design to compare the effects of diet. On the withdrawal of milk, we see a dramatic change in microbial populations. Specifically, in the abundances of *Lactobacillus*, a taxon that consumes plant-derived mono- and di-saccharides and milk sugars such as lactose but not complex milk sugars [28, 29]. Populations of saccharolytic microbes like *Prevotellaceae* increased from less than 0.3 % to more than 15 % of the total community, whose apparent comparative advantage for these substrates over *Bacteroidaceae* contributed to the concomitant decrease of *Bacteroidaceae* (Fig. 3). Increased relative abundances of β-glucanases, cellulases, xylanases, and cellobiases and associated downstream catabolic pathways all point to dramatic shifts in response to the compositional changes of diet, rather than exposure to new microbes from food (Fig. 4, Additional file 2: Figure S2). Of note, while the weaning feed contained antibiotics (tiamulin and chlortetracycline), the taxonomic composition of animals in this study resembled that of animals at similar ages in other studies with and without antibiotics [12, 13, 30], which indicates broad similarities in taxonomic and functional [31] composition, though antibiotic resistance may vary, in the microbiome of animals across different sites and production scenarios.

## Conclusions

Recent work has cast an important light on how the gut microbiome responds to changes in diet, using mouse models and human feeding trials [5, 32]. Here, we use a simple and highly relevant diet (milk) and a relevant and highly tractable animal model that resembles human digestion (pigs), whose diet composition is changed abruptly (from milk to near-adult feed) as a matter of course in rearing. The advantages in this model over other studies are evident in our level of detail at dissecting catabolic pathways using known dietary glycan structural data as a starting point. Our observations are in agreement with previous work in both pigs [12, 13] and human dietary studies investigating differences in animal and plant-based diets [33]. We also show the impact of abrupt and distinct changes in diet on the distal gut microbiome of pigs, at both a taxonomic and metagenomic level. Given that substrate availability was found to shape both taxonomic structure and enzymatic functional capacities, it is interesting to consider whether biogeographical variations in the gut microbiome and local variations in substrate availability may also be associated with similar taxonomic and functional differences. Future work may shed light in this respect on the role of the microbiome in digestion and whether specific pig milk oligosaccharides (PMOs) shape populations of microbes in pigs as HMOs shape the gut of human infants.

## Methods

### Animal experiments

All experiments involving animals were reviewed and approved by the University of California Davis Institutional Animal Care and Use Committee prior to beginning of the experiment (approval #17776). Throughout the study, all animals were housed in a controlled-access specific pathogen free facility at the University of California Davis. Three healthy Yorkshire/Hampshire adult pregnant multiparous sows from the University of California herd were selected for this study. Upon delivery, the infant pigs (*n* = 27) were cohoused with sows by litter and ear notched for individual identification and dosed with 50 mg spectinomycin and Penicillin G as benzathine and procaine (75,000 IU each), following standard husbandry practices for swine. The infant pigs were allowed to nurse freely until weaning after 21 days of age without creep feed and did not, to our knowledge, consume sow feed. Nursing pigs were removed from the sow and transferred to separate housing and fed a standard starter feed (Hubbard Feeds Mankato, MN USA) after 21 days of age. The weaning feed was oat-based and also contained 444 mg/kg chlortetracycline and 39 mg/kg tiamulin, while the sow diet was unmedicated. Animals were given ad libitum access to water and feed.

### Sampling

Fecal samples were collected rectally from each animal using a sterile cotton swab (Puritan Medical, Guilford, ME USA) wet with sterile phosphate buffered saline (pH 7.0) 1, 3, 5, 7, 14, 21, 28, 35, and 42 days after birth.

### Sequencing library construction

DNA was extracted from swabs using the Zymo Research Fecal DNA kit (ZYMO Research Irvine, CA USA) according to the manufacturer’s instructions. Extracted DNA was used as a template for PCR using barcoded primers to amplify the V4 region of the 16S rRNA gene as previously described [34, 35]. Briefly, the V4 domain of the 16S rRNA gene was amplified using primers F515 (5′-*NNNNNNNN***GT**GTGCCAGCMGCCGCGGTAA-3′) and R806 (5′-GGACTACHVGGGTWTCTAAT-3′), where the poly-*N* (italicized) sequence was an 8-nt barcode unique to each sample and a 2-nt linker sequence (bold). PCR amplification was carried out in a 15-μL reaction containing 1× GoTaq Green Mastermix (Promega, Madison, WI USA), 1 mM MgCl_2_ and 2 pmol of each primer. The amplification conditions included an initial denaturation step of 2 min at 94 °C, followed by 25 cycles of 94 °C for 45 s, 50 °C for 60 s, and 72 °C for 90 s, followed by a single final extension step at 72 °C for 10 min. Amplicons were pooled and purified using a Qiagen PCR purification column and submitted to the UC Davis Genome Center DNA Technologies Sequencing Core for paired-end library preparation, cluster generation, and 250-bp read sequencing on an Illumina MiSeq.

Quality-filtering of de-multiplexed reads was conducted as recommended [36] by removing low-abundance OTUs, to remove spurious reads and OTUs which result from artifacts of sequencing, and data was analyzed using QIIME 1.8.0 [37]. The 13_8 GreenGenes database release was used for open reference OTU picking and taxonomy assignment, and bacterial sequences were aligned using UCLUST [38]. To ensure even sequencing depth across samples, 7,000 sequences per sample were randomly subsampled for analysis of bacterial communities. Samples with fewer than 7,000 sequences were omitted. Alpha diversity estimates were computed for phylogenetic diversity (PD) and compared [39] by nonparametric two-sample *t* test with Bonferroni correction and 999 Monte Carlo permutations. Analysis of similarities (ANOSIM) distances were calculated using *compare_categories.py* using a weighted UNIFRAC distance matrix calculated by *beta_diversity.py*, both using QIIME [37]. Family-level taxa abundances were used in STAMP [40] to generate principal component analyses (PCA). All 16S rRNA sequencing data is publicly available in QIITA (http://qiita.microbio.me).

### Metagenome sequencing

Total genomic DNA was extracted from fecal samples with the ZYMO Research Fecal DNA Extraction kit according to manufacturer’s instructions and prepared using the Illumina MiSeq v3 Reagent Chemistry for whole genome shotgun sequencing of multiplexed 150-bp libraries at the University of California Davis Genome Sequencing Core (http://dnatech.genomecenter.ucdavis.edu). Samples used for metagenomic sequencing were selected from one animal from each litter, sampled before and after weaning, on day 14 (nursing, *n* = 3) and day 35 (weaned, *n* = 3). Barcoded sample libraries were pooled and sequenced across triplicate, single-read sequencing runs to reach adequate sequencing depth for fecal samples [41] and prevent run-to-run variation. FASTQ files from each run were de-multiplexed, quality filtered, and trimmed to 150 bp, and then reads for each sample were pooled from the three runs, yielding 15–20 million reads per sample, and submitted to the Metagenomics Rapid Annotation using Subsystem Technology (MGRAST) pipeline for analysis [42], which removes host genomic DNA reads and duplicate reads, bins 16S rRNA reads, and functionally classifies remaining reads by predicted protein sequence using several different schemes. All metagenomic sequencing data is publicly available on the MGRAST website (http://metagenomics.anl.gov). Subsystem classified reads were functionally classified and normalized in MGRAST and compared between groups by multiple *t* test using GraphPad Prism 6 (GraphPad Software Inc, La Jolla, CA).

## Additional files

Additional file 1: Figure S1. Relative abundance of *Prevotellaceae* increases at weaning. Barchart, grouped and colored by sampling date, showing the relative abundance of *Prevotellaceae* over time. Additional file 2: Figure S2. Normalized abundances of predicted genes involved in (A) milk glycan or monomer consumption and (B) xylose or arabinose release and catabolism. All differences between diets shown (blue, nursing; red, weaned) were significant (*p* < 0.01), unless indicated with an asterisk, where *p* < 0.05.
