# Supplementary figures and images for: Diet shapes the gut microbiome of pigs during nursing and weaning
Source: Microbiome. 2015 Jul 1;3:28. doi: 10.1186/s40168-015-0091-8 (PMC4499176; doi:10.1186/s40168-015-0091-8)

## Small Populations of *Prevotella* Shift Only at Weaning

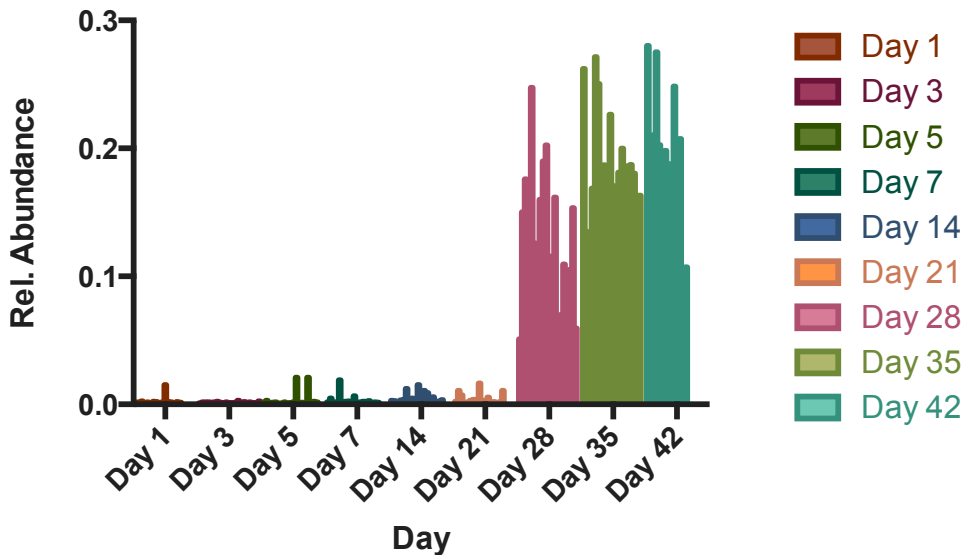

Supplement: Additional file 1: Figure S1. — Relative abundance of Prevotellaceae increases at weaning. Barchart, grouped and colored by sampling date, showing the relative abundance of Prevotellaceae over time. [file 40168_2015_91_MOESM1_ESM.pdf]
