# Supplementary material for: Diet shapes the gut microbiome of pigs during nursing and weaning
Source: Microbiome. 2015 Jul 1;3:28. doi: 10.1186/s40168-015-0091-8 (PMC4499176; doi:10.1186/s40168-015-0091-8)
Supplement: Additional file 2: Figure S2. — Normalized abundances of predicted genes involved in (A) milk glycan or monomer consumption and (B) xylose or arabinose release and catabolism. All differences between diets shown (blue, nursing; red, weaned) were significant (p < 0.01), unless indicated with an asterisk, where p < 0.05. [file 40168_2015_91_MOESM2_ESM.pdf]

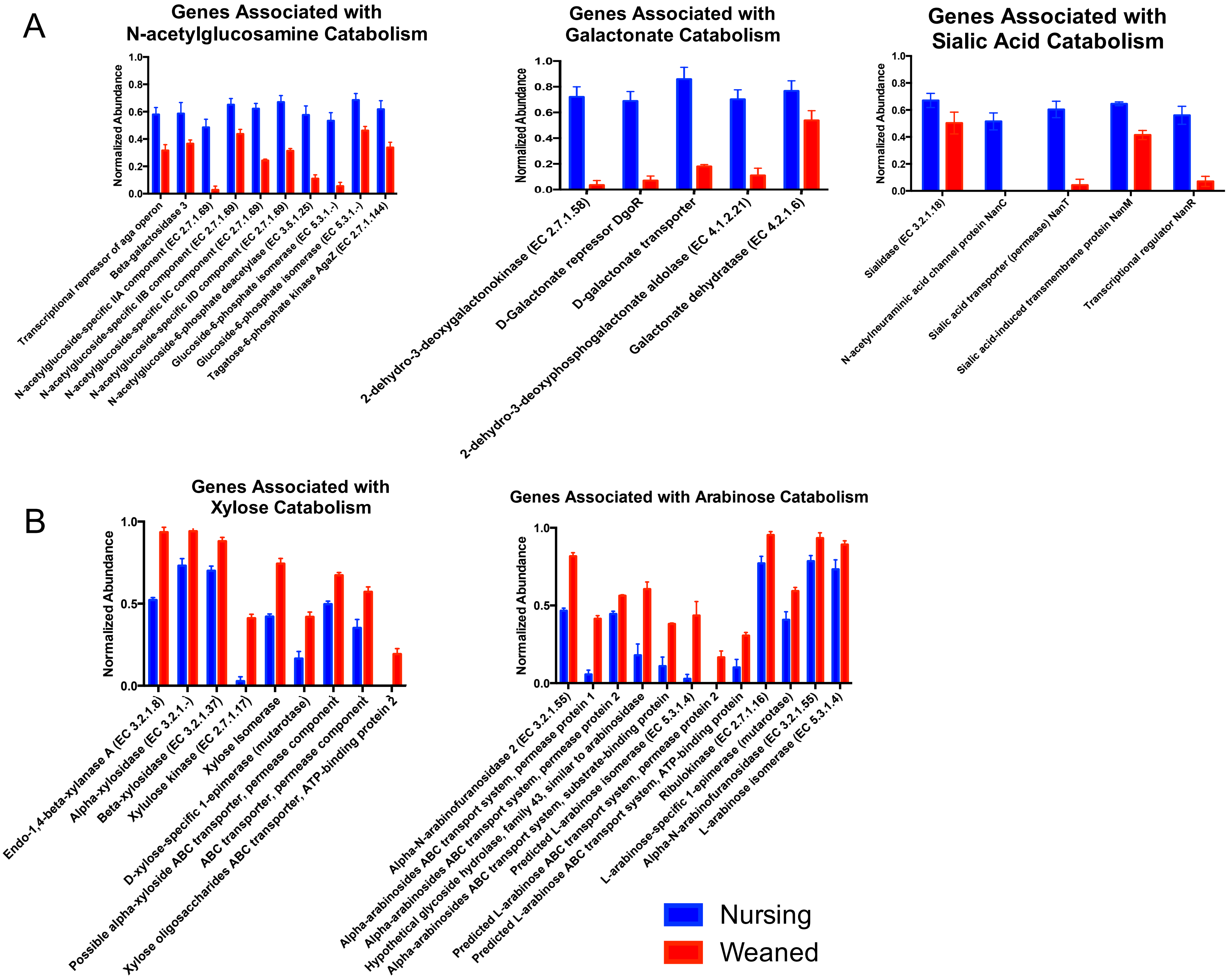
A
Genes Associated with N-acetylglucosamine Catabolism
Genes Associated with Galactonate Catabolism
Genes Associated with Sialic Acid Catabolism
Normalized Abundance
Transcriptional repressor of aga operon
Beta-galactosidase 3
N-acetylglucosamine-specific IIA component (EC 2.7.1.69)
N-acetylglucosamine-specific IIB component (EC 2.7.1.69)
N-acetylglucosamine-specific IIC component (EC 2.7.1.69)
N-acetylglucosamine-specific IID component (EC 2.7.1.69)
N-acetylglucosamine-6-phosphate deacetylase (EC 3.5.1.25)
Glucoside-6-phosphate isomerase (EC 5.3.1.-)
Glucoside-6-phosphate isomerase (EC 5.3.1.-)
Tagatose-6-phosphate kinase AgaZ (EC 2.7.1.144)
2-dehydro-3-deoxygalactonokinase (EC 2.7.1.58)
D-Galactonate repressor DgoR
D-galactonate transporter
2-dehydro-3-deoxyphosphogalactonate aldolase (EC 4.1.2.21)
Galactonate dehydratase (EC 4.2.1.6)
Sialidase (EC 3.2.1.18)
N-acetylneuraminic acid channel protein NanC
Sialic acid transporter (permease) NanT
Sialic acid-induced transmembrane protein NanM
Transcriptional regulator NanR
B
Genes Associated with Xylose Catabolism
Genes Associated with Arabinose Catabolism
Endo-1,4-beta-xylanase A (EC 3.2.1.8)
Alpha-xylosidase (EC 3.2.1.-)
Beta-xylosidase (EC 3.2.1.37)
Xylulose kinase (EC 2.7.1.17)
Xylose Isomerase
D-xylose-specific 1-epimerase (mutarotase)
Possible alpha-xyloside ABC transporter, permease component
ABC transporter, permease component
Xylose oligosaccharides ABC transporter, ATP-binding protein 2
Alpha-N-arabinofuranosidase 2 (EC 3.2.1.55)
Alpha-arabinosides ABC transport system, permease protein 1
Alpha-arabinosides ABC transport system, permease protein 2
Hypothetical glycoside hydrolase, family 43, similar to arabinosidase
Alpha-arabinosides ABC transport system, substrate-binding protein
Predicted L-arabinose isomerase (EC 5.3.1.4)
Predicted L-arabinose ABC transport system, permease protein 2
Predicted L-arabinose ABC transport system, ATP-binding protein
Ribulokinase (EC 2.7.1.16)
L-arabinose-specific 1-epimerase (mutarotase)
Alpha-N-arabinofuranosidase (EC 3.2.1.55)
L-arabinose isomerase (EC 5.3.1.4)
Nursing
Weaned
